# Attractiveness and gender dynamics in surgical specialties: a comparative analysis of French medical graduates (2017–2022)

**DOI:** 10.1186/s12909-024-05174-y

**Published:** 2024-02-27

**Authors:** Saadé Saadé, Arnaud Delafontaine, Johann Cattan, Doris Celanie, Gabriel Saiydoun

**Affiliations:** 1 Department of Cardiac Surgery, Hôpitaux Universitaires de Strasbourg, Nouvel Hôpital Civil, 67000 Strasbourg, France; 2 Université Libre de Bruxelles, Faculté de Médecine, Route de Lennik, Bruxelles, 1070 Belgium; 3 Department of Cardiac Surgery, CHU de Bordeaux, Place Amélie Raba Léon, Bordeaux, 33000 France; 4 Université des Antilles, 97100 Pointe-à-Pitre, Guadeloupe France; 5 Department of cardiac surgery, Pitié-Salpêtrière, Bld Vincent Auriol, 75013 Paris, France

**Keywords:** Attractiveness, Gender dynamics, Surgical specialties, French medical graduates, Residency programme

## Abstract

**Background:**

French medical graduates undertake a national examination at the end of their studies with a subsequent national ranking. Specialty is then chosen by each candidate according to their ranking. This study aims to describe the attractiveness of surgical specialties and the evolution of the male-female distribution among French medical graduates (FMG) from 2017 to 2022.

**Methods:**

Our database included the candidates’ ranking, sex and choice of specialty from 2017 to 2022. It included all French medical graduates from 2017 to 2022 and all French medical schools. A linear regression was performed to predict future trends. Dependent variables were mean rankings and the percentage of women. The independent variable was year of application. A Pearson correlation was performed to examine any relationship with mean workweek.

**Results:**

A total number of 5270 residents chose a surgical programme between 2017 and 2022. The number of residents who were assigned their desired surgical programme held stable at 878 surgical residents per year. Plastic and reconstructive surgery remained the most frequently chosen surgical programme. Thoracic and cardiovascular surgery was the least frequently chosen surgical programme between 2017 and 2022. The mean ranking for a candidate choosing a surgical programme rose significantly by 9% from 2017 to 2022 (*p* < 0.01). Neurosurgery exhibited the greatest fall as a surgical specialty as its rankings decreased by 163.6% (*p* < 0.01). Maxillo-facial surgery was the only specialty with a statistically significant increase in its rankings by 35.9% (*p* < 0.05). The overall proportion of women was 51.1%. Obstetrics-and-gynecology was the highest represented specialty among female candidates, with a mean of 83.9% of women. Orthopedic surgery was the lowest represented, being composed of a mean of 28.6% of women. The number of female surgical residents increased significantly over the six-year period, by 7.6% (*p* < 0.01).

**Conclusions:**

More and more medical school graduates decide not to choose surgery for their residency programme. Some specialties continue to be attractive while many are losing their appeal. While there does appear to be progress towards gender equity, further investigation is necessary to assess its actual implementation.

## Background

In France, medical studies are organised around two major competitive examinations. A first examination after the first year of university determines a candidate’s entry into medical school. Then, after completing medical school, each candidate chooses a medical specialty and a region of practice based on their ranking national ranking examination (NRE or *Epreuves Classantes Nationales)* covering all medical specialties, which is held over the course of 3 days*.* Following the examination, candidates express their wish for a given specialty and city. Candidates are then matched with a specialty and city based on their ranking in the examination. For example, the candidate ranking third chooses after the candidates ranked first and second, out of a limited pool of positions and cities, as determined by the Ministry of Health on a yearly basis. Thus, candidates with a higher ranking tend to choose less attractive specialties. In 2017, a new reform modified the general surgery/subspecialty programme. Since then, candidates choose their surgical subspecialty directly from thirteen available programmes without the need of a general surgery programme: digestive and visceral surgery, Ear nose and throat (ENT), maxillo-facial surgery, neurosurgery, Obstetrics and Gynecology (OB-GYN), ophthalmology, oral surgery, orthopedic surgery and traumatology, pediatric surgery, plastic and reconstructive surgery, thoracic and cardiovascular surgery, vascular surgery, and urology.

In primary care, these decisions have had major implications on physician supply and demand, research and productivity [1]. Various factors are shaping the landscape of the physician workforce, with major disparities between geographical locations, in both medicine and surgery. However, three main factors consistently play a role in candidates’ choices: the ability to enjoy a ‘controllable lifestyle’, personal interest in a particular type of medical activity and intellectual challenge [2, 3].

Interest in surgical careers has been declining globally over the past two decades. In Canada, surgical applications fell from 24.7% in 1998 to 17.2% in 2016 [4]. The number of residents applying for a surgical programme in the United States has dropped significantly over the past two decades, with 25% of general surgery positions now being filled by international medical graduates (IMG). The situation in Great Britain is the same: ad hoc testing has shown that medical students do not consider a career in surgery to be appealing [5].

Despite this decline in enthusiasm for surgical training programmes, the proportion of female surgical residents has increased significantly over the past 10 years in many countries, including Canada and the USA [6–8]. This is partly explained by the parallel increase in the number of female medical students, with women making up more than 50% of incoming US medical students [9–11]. However, discrepancies among surgical specialties exist, with several specialties remaining underrepresented, including orthopedic surgery, plastic surgery and urology [12].

Only limited information on changes in surgical specialties in France is available, and data describing gender distribution in French surgical residents remain scant, especially after the 2017 reform.

Therefore, in this study, we aimed to accurately describe the attractiveness of surgical specialties and their gender distribution among French surgical residents during the 2017–2022 period.

## Methods

### Data collection

Data was obtained from official public records published annually by the Ministry of Health, specifying the sex, date of birth, ranking, city and specialty of each French Medical Graduate (FMG) [13–18].

The mean workweek for each specialty was determined by a public survey conducted by the National Union of Residents (*ISNI: InterSyndicale Nationale des Internes*) in 2021 [19]. This initiative aimed to implement the 2019 decision of the European Union, which formally mandates its member states to ensure that hospitals establish a system for monitoring the duration of daily working hours.

### Attractiveness index (AI)

This index was first introduced by the Ministry of Health’s Directorate of Research, Studies, Evaluation and Statistics (*DREES*) in 2014 [20]. It is calculated for each subspecialty:

$$\textrm{Attractiveness}\ \textrm{Index}=\left(\textrm{SOR}-\textrm{SORIP}\right)/\left(\textrm{SOR}\textrm{IR}-\textrm{SORIP}\right)$$

- SOR: sum of obtained rankings for a subspecialty.
- SORIP: sum of obtained rankings if preferred. It represents the sum of rankings for a given subspecialty if it was unanimously preferred by the top-ranking candidates.
- SORIR: sum of obtained rankings if rejected by all the candidates.

Consequently, specialties with an index closest to 0 are the most attractive (SOR closer to SORIP), and those with an index closest to 1 are the least attractive (SOR closest to SORIR). An attractive specialty usually has an AI< 0.2, while less competitive specialties have an AI> 0.4.

### Statistical analysis

We used various metrics to assess trends in surgical position choices, and descriptive statistics to evaluate gender distribution. Considering that all personal information was de-identified and that all the data analysed are publicly available, approval from the research ethics board was not necessary for this study. Because specialty choice takes place in June of every given year, the pre COVID-19 period was defined as 2017–2019, and the post COVID-19 as 2020–2022. Continuous variables were expressed as median and interquartile range (IQR), or mean and standard deviation (SD). T-tests were performed to compare continuous variables. Temporal trends for rankings and the weighted mean percentage of women in each surgical subspecialty was assessed with a linear regression. The independent variable was time (i.e., the year of application) and the dependent variables were subspecialty mean ranking and percentage of women. We reported linear regression results in *p*-values to assess significance. A *p*-value of less than or equal to 0.05 (two-sided) was considered significant. Additionally, we thought it interesting to correlate the mean AI and gender distribution of each subspecialty with the mean workweek (in hours per week) of each subspecialty with a Pearson correlator(r). We used Prism database (GraphPad Prism 5; Graph Pad Software, San Diego, California) for all analyses.

## Results

**Fig. 1:**
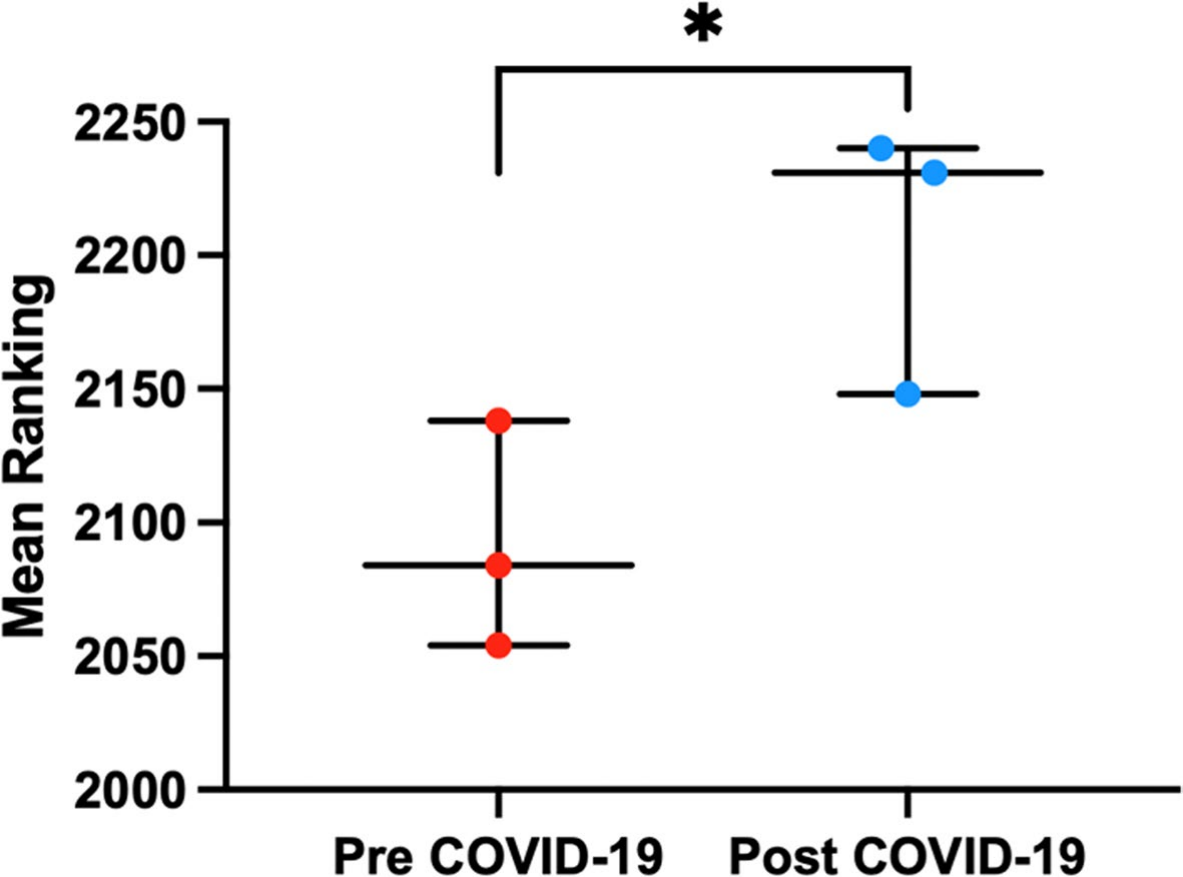
Mean ranking of all combined surgical specialties before the pandemic (Red: Pre COVID-19; 2017–2019) and after the pandemic (Blue: Post COVID-19; 2020–2022)

During the 2017–2022 period, a total of 52,951 FMG chose a medical specialty after passing the NRE, with a mean of 8825 (*SD =* 314) applicants/year. Of these, 5270 FMG chose surgical specialties. No surgical position remained unfilled. Overall, the mean ranking of all combined surgical specialties rose significantly by 9.0%. (*p* < 0.01) (Table 1). Additionally, the mean ranking after the COVID-19 pandemic was significantly higher than before (*p* < 0.05) (Fig. 1).

**Table 1:** Mean ranking of all combined surgical specialties in France from 2017 to 2022

Year	Mean ranking	SD
2017	2054	1052
2018	2138	1075
2019	2084	1085
2020	2148	1193
2021	2231	1200
2022	2240	1267

From 2017 to 2022, ophthalmology, and plastic and reconstructive surgery were the most competitive surgical specialties (Table 2) and the most competitive of all medical and surgical specialties (Data not shown). Thoracic and cardiovascular, and vascular surgery were the least popular surgical specialties with the highest mean ranking at 3961 (*SD* = 361) and 3776 (*SD =* 235) respectively.

**Table 2:** Mean ranking by surgical specialty in France from 2017 to 2022

	Plastic and reconstructive surgery	Ophthalmology	Maxillo-facial surgery	ENT	Urology	Orthopedic surgery	OB-GYN	Neurosurgery	Oral surgery	Pediatric surgery	Visceral and digestive surgery	Vascular surgery	Thoracic and cardiovascular surgery
2017	665	591	1982	1318	2053	1958	2582	1186	2582	3118	3223	3561	3746
2018	771	630	1559	1663	1920	1850	2900	2137	1989	3212	3532	3915	3718
2019	532	593	1443	1490	2165	2248	2771	2152	3950	2596	3245	3438	3501
2020	347	636	1003	1492	2104	2227	2762	2436	3163	2760	3486	3751	4158
2021	404	639	1372	1411	2465	2799	2799	2796	3109	3016	3549	3987	4193
2022	558	631	1272	1500	2337	2641	2641	3127	3424	3037	3832	4004	4454
Mean Ranking	546	620	1438	1479	2174	2168	2742	2305	3036	2956	3477	3776	3961
SD	158	21	326	114	197	237	114	668	679	232	224	235	361
*P*-Value			< 0.05		< 0.01	< 0.01		< 0.01			< 0.05	< 0.05	< 0.01

The rankings of digestive (*p* < 0.05) and vascular surgery (*p* < 0.01) rose by 18.9 and 12.4%, respectively, while those of orthopedic surgery, urology, and thoracic and cardiovascular surgery increased by 12.5% (*p* < 0.01), 13.83% (*p* < 0.01) and 18.9% (*p* < 0.01), respectively. Neurosurgery saw the greatest decline as a surgical specialty, with its ranking rising by 163.9% (*p* < 0.01).

The rankings of several surgical specialties remained relatively unchanged: plastic and reconstructive surgery, ophthalmology, ENT, OB-GYN, oral surgery, and pediatric surgery.

Maxillo-facial surgery was the only specialty to see a statistically significant improvement in its ranking: 35.9% (*p* < 0.05).

Only four surgical specialties were considered attractive with a mean attractiveness index **(**AI) < 0.2: plastic and reconstructive surgery 0.06 (*SD* = 0.01), ophthalmology 0.08 (*SD* = 0.006), maxillo-facial surgery 0.16 (*SD* = 0.03) and ENT 0.18 (*SD* = 0.01) (Table 3). Vascular surgery 0.40 *(SD =* 0.006), and thoracic and cardiovascular surgery 0.42 (*SD =* 0.02) had the highest mean AI of all surgical specialties (i.e., considered unattractive by applicants).

**Table 3:** Mean attractiveness index by surgical specialty in France from 2017 to 2022

Plastic and reconstructive surgery	Ophthalmology	Maxillo-facial surgery	ENT	Urology	Orthopedic surgery	OB-GYN	Neurosurgery	Oral surgery	Pediatric surgery	Visceral and digestive surgery	Vascular surgery	Thoracic and cardiovascular surgery
0.06	0.08	0.16	0.18	0.26	0.27	0.32	0.27	0.35	0.34	0.37	0.4	0.42

In 2021, the National Union of Residents (*ISNI*) reported a mean workweek for surgical specialties of 69.5 hours. Several surgical specialties had a workweek of over 70 hours (Table 4): neurosurgery (82.24 hours), digestive surgery (77.72 hours), orthopedic surgery (77.29 hours), thoracic and cardiovascular surgery (76.03 hours), urology (76.03 hours), vascular surgery (74 hours) and pediatric surgery (73.42 hours). No surgical specialty respected the 48-hour workweek limit set by the Ministry of Health in 2015.

**Table 4:** Mean workweek in hours by surgical subspecialty as reported by the National Union of Residents (*ISNI*) in 2021

Surgical subspecialty	Mean workweek (h)
Plastic and reconstructive surgery	68.52
Ophthalmology	51.87
Maxillo-facial surgery	67.43
ENT	65
Urology	76.03
Orthopedic surgery	77.29
OB-GYN	69.64
Neurosurgery	82.24
Oral surgery	54.38
Pediatric surgery	73.42
Digestive surgery	77.72
Vascular surgery	74
Thoracic and Cardiovascular surgery	76.03

*ENT* Ear, nose and throat, *OB-GYN* Obstetrics and gynecology

As we observed some discrepancies between specialties’ mean workweek, we decided to perform analyses to determine whether there was a relationship between surgical specialty choice and mean workweek. We found no linear correlation between the mean AI and the workweek (in hours) of surgical specialties: *r* = 0.45, *p* = 0.12.

To explore an additional aspect of the surgical landscape, we decided to examine the male-female distribution (Table 5).

**Table 5:** Percentage of female surgical applicants (per specialty) in France from 2017 to 2022

Year	Plastic and reconstructive surgery	Ophtalmology	Maxillo-facial surgery	ENT	Urology	Orthopaedic surgery	OB-GYN	Neurosurgery	Oral surgery	Paediatric surgery	Visceral and digestive surgery	Vascular surgery	Thoracic and Cardiovascular Surgery
2017	37%	40%	50%	44%	40%	29%	78%	24%	25%	54%	43%	35%	40%
2018	27.6%	38.1%	44.4%	51.9%	38.7%	18.9%	80.5%	36%	50%	59.1%	38.7%	21.4%	32%
2019	35.7%	38.8%	61.5%	51.9%	36.1%	30%	88.3%	40%	33.3%	77.3%	41%	27%	20%
2020	32.1%	43.7%	40%	48.1%	31.1%	24.8%	80.4%	24%	58.3%	65.2%	57%	44.4%	44%
2021	28.6%	37.4%	50%	55.4%	43.5%	39.8%	86.9%	32%	33.3%	80.8%	51.8%	53.6%	40%
2022	28.6%	41%	42.3%	56.3%	39.1%	29.1%	89.2%	63%	57.1%	62.1%	50.6%	68%	60%
Mean percentage	31.6%	39.9%	48%	51.3%	38.1%	28.6%	83.9%	36.4%	42.8%	66.4%	47%	41.5%	39.3%
SD	4	2	7	4	4	6	4	10	10	10	7	17	13
*P*-Value				< 0.1			< 0.01				< 0.01	< 0.01	< 0.05

Over the six-year period, females represented 51.1% of all surgical applicants. Women remained under-represented primarily in plastic and reconstructive surgery 31.6% (*SD =* 4), and orthopedic surgery 28.6% (*SD =* 6). By contrast, they represented 83.9% (*SD =* 4) of candidates for OB-GYN, and 66.4% (*SD =* 10) of those in pediatric surgery.

**Fig. 2:**
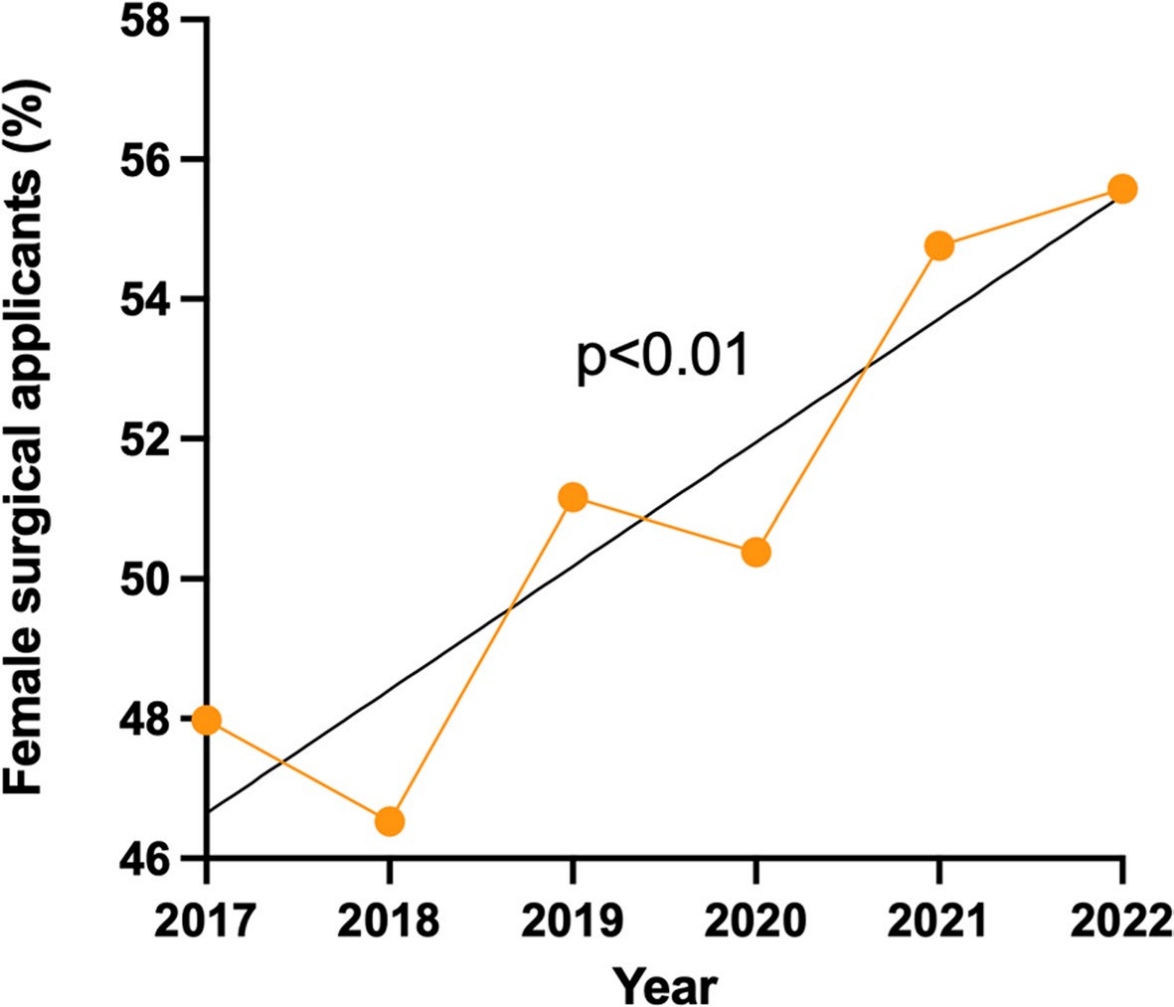
Percentage of female surgical applicants (all surgical specialties combined) in France from 2017 to 2022

Of note, the overall percentage of women increased by 7.6% (*p* < 0.01) with women representing 55.6% of all surgical applicants in 2022 (Fig. 2).

This was very apparent in several specialties: + 33% in vascular surgery (*p* < 0.01) + 20% in thoracic and cardiovascular surgery (*p* < 0.05) + 12.3% in ENT (*p* < 0.1) + 11.2% in OB-GYN (*p* < 0.01), and + 6.4% in digestive surgery (*p* < 0.01).

There was no significant change in the number of women working in pediatric surgery neurosurgery, oral surgery, orthopedic surgery, urology, maxillo-facial surgery, ophthalmology or plastic and reconstructive surgery.

Moreover, there was no interaction between gender distribution and the average workweek (*r* = − 0.21, *p* = 0.5).

## Discussion

In this study, we describe the choices of surgical specialties in a French population of 52,951 FMG, and the male-female distribution in each subspecialty. We demonstrated that surgery is becoming a less attractive field of medicine, and especially neurosurgery, thoracic and cardiovascular surgery, and orthopedic surgery. We also showed that there is a clear overall increase in numbers of female surgeons, especially in thoracic and cardiovascular surgery, ENT, and vascular surgery. To our knowledge, this is the first study weighting the trends of each surgical specialty in terms of attractiveness and gender in France since the 2017 reform.

No surgical position remained vacant, and surgery remains a relatively attractive specialty in France. However, we have shown that there has been a decline in the number of highly ranked students pursuing a career in surgery. Bartlett et al., among others, suggest that this is an international trend and that it will most likely continue [21], resulting in surgical positions going unfilled unless corrective action is taken. This trend could be due to changing priorities, with students tending to prioritise a more ‘controllable lifestyle’ as widely described in French and North American literature [3, 4, 22, 23]. A ‘controllable lifestyle’ is defined by the physician’s control of time spent on professional responsibilities [3]. However, control of time alone doesn’t fully account for a candidate’s specialty choice. Indeed, we showed that surgical specialties such as plastic surgery, maxillo-facial surgery and ENT remain very competitive (low AI) despite being traditionally classified in the ‘uncontrollable lifestyle’ [22] category with a high number of working hours. These specialties may be doing a better job engaging with medical students early in their career, have the potential of being well remunerated and are typically less responsible for emergency or critically ill patients. Geographical location could potentially introduce a confounding factor, but given that nearly all cities provide access to any surgical subspecialty, we believe the decision is primarily influenced by the candidate’s ranking [24]. The COVID-19 pandemic may have had a significant impact on the decisions made by individuals pursuing medical careers. Specifically, the pandemic altered the exposure and shadowing experiences of medical students, particularly affecting surgical disciplines. The closure of operating rooms to accommodate the surge in patients further contributed to this shift. Additionally, the pandemic prompted a reassessment of healthcare priorities on a global scale. Although studies have demonstrated this phenomenon in various regions [25, 26], there remains a dearth of such research in the context of France.

A recruitment shortfall in surgery and demanding surgical specialties such as neurosurgery or thoracic and cardiovascular surgery, similar to trends in other nations, presumably will affect rural areas most severely [27–29]. It has been shown in North American literature that the consequences of a shortage of surgeons are dire [27, 29], particularly for trauma cases. An impending crisis appears inevitable unless active promotion is taken to ensure the survival of the profession. Thomas et al. showed that part of the solution lies in grassroots events such as career and skills days with basic skills simulations [30]. We encourage such events in France, especially for specialties in a critical situation.

Thoracic and cardiovascular surgery is one example of a career in a state of emergency. Despite it previously being one of the most competitive fields, our estimates suggest that it will be the first surgical subspecialty to experience a downturn in France. This mirrors similar recruitment problems in the UK [31, 32], Canada [33] and the United States [34]. Thoracic and cardiovascular surgery is a small but physically and psychologically challenging specialty. It requires methodical teamwork and has a non-negligible fatality rate. The factors leading candidates to pursue this profession differ from other surgical specialties: the ability to save lives, intellectual challenge and the skilled nature of the surgery were the most attractive factors in a UK survey [32]. Early exposure to a positive mentor [34] and adequate planning by health authorities to ensure long-term job security are essential to safeguard perennity of the profession.

Work-life balance is very important for candidates when selecting a subspecialty. A satisfactory work-life balance relies heavily on the mean workweek. Surprisingly, our study failed to demonstrate an effect of workweek on the choice of surgical specialty. This may be due partly to a selection bias: for surgical candidates, working long hours may not be as much of a discriminating factor as it is in primary care specialties. The issue of mental health in the field of surgery is a growing concern, as highlighted by Chati et al. [35]. According to their findings, French surgery residents experience higher rates of Burn-Out syndrome compared to their medical counterparts. Specifically, they exhibit elevated levels of depersonalization and lower rates of personal achievement. With an increasing number of medical students prioritizing mental health in their choice of specialty, there is a potential trend of individuals opting against pursuing surgery due to these mental health challenges. One other explanation might be the salary that comes with working longer hours. Physician incomes are low in France [36], so residents may be willing to work additional hours to compensate for low pay, since they receive extra remuneration on a per-duty basis [36]. A more in-depth inquiry is needed to establish the elements that French surgical residents consider important for a good work-life balance.

Gender also plays a role in the choice of surgical specialty. In this study, we showed that most OB-GYN candidates are female, while most orthopedic surgery candidates are male. The French surgical landscape is generally well balanced, and the number of women pursuing a career in surgery has risen gradually over the years. In 2022, women held most surgical positions (55.6%), and we have shown that this trend will likely continue. This trend may be due to the feminisation of medicine overall, since 64% of medical students in France are women as shown in multiple French governmental reports. Several traditionally male-dominated specialties, such as neurosurgery, and thoracic and cardiovascular surgery, are now seeing a more balanced gender distribution. However, senior members in these specialties remain predominantly male [37, 38], and efforts should focus on implicit bias [39] and women in surgical academia.

### Study limitations

Our study’s primary advantage is that it encompassed all future applicants in surgery. However, it has several limitations. First, to assess a specialty’s competitiveness, it focused on the mean ranking and Attractiveness Index as determined by the NRE. The NRE is skewed in that it is a purely theoretical examination with no surgical element. Evaluation bias might exist and future reforms of the NRE incorporating practical skills and surgical placements will paint a clearer picture of surgical competitiveness. Second, it only had a limited set of factors that could explain disparities between surgical specialties. Geographical location seems like an important factor influencing specialty choice that was not analysed in our study. A composite assessment including criteria such as work-life balance, salary, geographical location, presence of a positive role model and prestige would have provided a better understanding of the underlying motivators in a candidate’s choice of specialty. Finally, to better adapt surgery teaching to French medical graduates’ requirements, a national prospective study is needed to address medical students’ changing perception of surgery from first to last year of medical school.

## Conclusion

This study’s findings indicate a growing decline in interest in surgery in France. The results vary across surgical specialties, with some being affected more significantly than others. This complex process requires a comprehensive evaluation due to its multifactorial nature. The implications for public health, society and surgical training are significant, and could lead to an imbalance between medical supply and demand. Our data suggest that there is a likelihood of deterioration in the foreseeable future. Our objective is to raise awareness and encourage necessary prompt corrective action. Additionally, there is a noticeable trend of greater representation of women in surgical specialties in France, which is a promising development towards achieving gender parity. However, this phenomenon must be examined thoroughly to ensure the adequate inclusion of women in the surgical field.

## Data Availability

The dataset supporting the conclusions of this article is available publicly and in the private repository:https://www.dropbox.com/scl/fi/d75dh1s3yhfkvfx8jw4tz/Data-Surgical-specialties.xlsx?rlkey=068u64lxlqh445ndcsrrlkxm9&dl=0.

## Abbreviations

AI: Attractiveness index
ENT: Ear nose and throat
FMG: French Medical Graduate
NRE: National ranking examination
OB-GYN: Obstetrics and Gynecology
SOR: Sum of obtained rankings
SORIP: Sum of obtained rankings if preferred
SORIR: Sum of obtained rankings if rejected
